# Supplementary figures and images for: Mapping young people’s journeys through mental health services: A prospective longitudinal qualitative study protocol
Source: PLoS One. 2023 Jun 13;18(6):e0287098. doi: 10.1371/journal.pone.0287098 (PMC10263348; doi:10.1371/journal.pone.0287098)

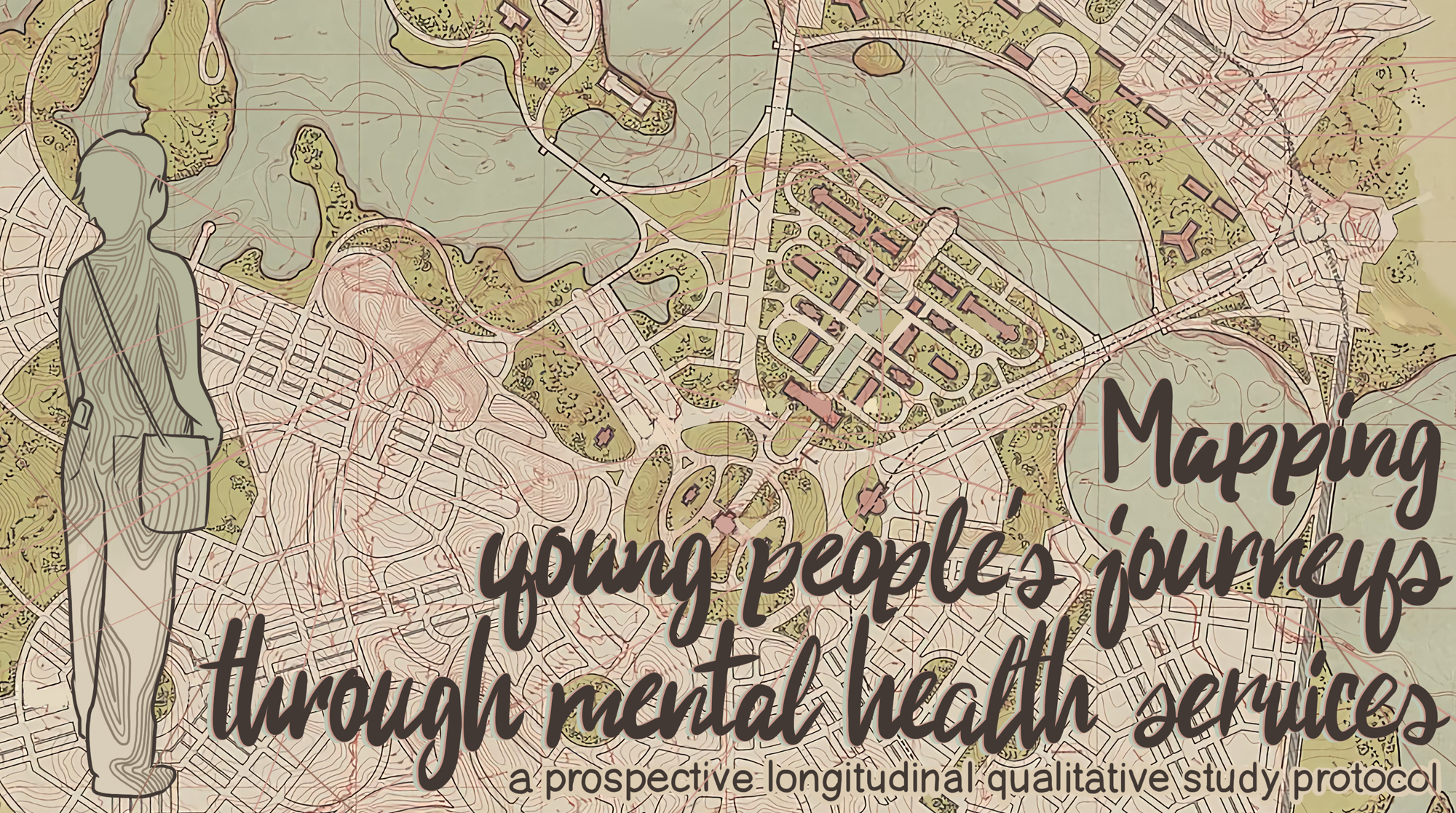

Supplement: S1 Graphical abstract — (JPG) [file pone.0287098.s002.jpg]
